# Diels-Alder Reactions of 12-Hydroxy-9(10→20)-5aH-abeo-abieta-1(10),8(9),12(13)-triene-11,14-dione

**DOI:** 10.3390/molecules18066969

**Published:** 2013-06-14

**Authors:** George Majetich, Yong Zhang, Xinrong Tian, Ge Zou, Yang Li, Yangyang Wang, Shougang Hu, Eric Huddleston

**Affiliations:** Department of Chemistry, University of Georgia, Athens, GA, 30602, USA

**Keywords:** Diels-Alder reaction, cascade process, one-pot reaction, Lewis acid catalysis

## Abstract

12-Hydroxy-9(10→20)-5aH-abeo-abieta-1(10),8(9),12(13)-triene-11,14-dione (quinone **2**) served as the dienophile in numerous intermolecular Diels-Alder reactions. These cycloadditions were conducted either thermally (including microwave heating) or with Lewis acid activation. While most dienes reacted with quinone **2** in good chemical yield, others were incompatible under the experimental conditions used.

## 1. Introduction

In a Fall 1974 invited lecture before the Pittsburgh Section of the ACS, Professor Samuel Danishefsky stated, reporting on his group’s progress toward a synthesis of vernolepin, that: “A synthesis cannot be considered truly elegant if it does not contain at least one Diels-Alder reaction.”

**Scheme 1:**
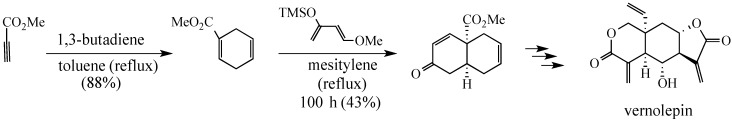
Danishefsky *et al.*’s synthesis of vernolepin.

The transformations being cited are shown in Scheme 1 and are discussed in [1].

Since its discovery in 1928 by Otto Diels and Kurt Alder [2], the cycloaddition of a diene with a dienophile has become a powerful method to construct a cyclohexene ring, thanks to its remarkable regioselectivity, syn stereospecificity, and the capability to create as many as four chiral centers in a single transformation [3,4]. The past fifty years have seen Diels-Alder reactions expand from making cyclohexene rings, either inter- or intramolecularly [5,6,7], to a useful means to prepare six-membered ring heterocycles via the *hetero*-Diels-Alder reaction [8,9,10,11,12,13], and as a practical way to prepare complex polycyclic frameworks via the *homo*-Diels-Alder reaction [14,15], and for creating asymmetry [16,17,18]. This explosion of activity is reflected in the large number of books and reviews focused on the advancements made in Diels-Alder reactions [19,20,21,22,23,24,25].

One way for chemists to devise the most efficient synthetic route possible for a given compound is to recognize when two or more transformations can be achieved under the same reaction conditions without isolation of the intermediate product(s) [26,27,28,29,30,31,32]. Indeed, the more sequential transformations that can occur, the fewer the steps needed to achieve a given total synthesis, and hence the more efficient the synthesis is. Such transformations have been widely studied and are described in the literature either as a cascade, [33] domino, [34] tandem, [35] consecutive, [36] or as an one-pot reaction. It is not surprising that Diels-Alder reactions are particularly useful for multiple bond formations. 

**Scheme 2:**
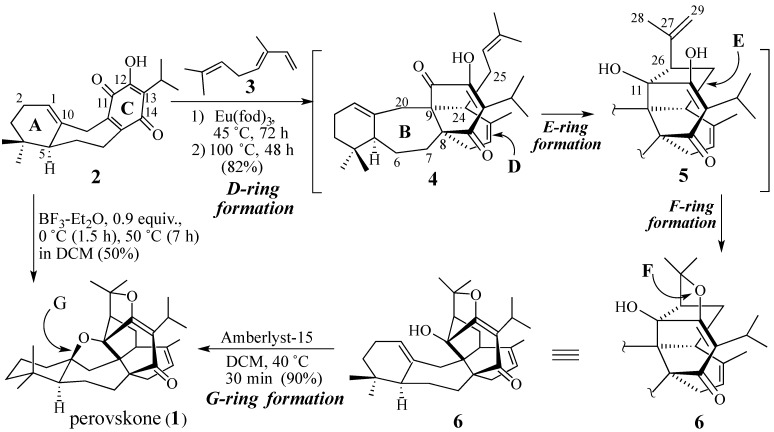
The cascade-based total synthesis of perovskone.

In 1994 we achieved a 16-step synthesis of the triterpene (±)-perovskone (**1**) [37] that featured a cascade process in which three rings and five stereocenters of the product were created in a single operation (Scheme 2) [38].

Treatment of racemic benzoquinone **2** and trans-α-ocimene (**3**) with the mild Lewis acid *tris*-(6,6,7,7,8,8,8-heptafluoro-2,2-dimethyl-3,5-octanedionato)europium,* i.e.*, Eu(fod)_3_, [39,40,41] at 45 °C for 72 h, followed by heating at 110 °C for 48 h, produced tertiary alcohol **6** in 82% yield. The first step in the formation of **6** is a Diels-Alder reaction which created the D-ring with the requisite relative configurations at C-8, C-9, and C-24 (cf. adduct **4**). The use of ocimene as the diene component also facilitated the formation of the C-11, C-26 σ-bond, the C-11 chiral center as a result of an intramolecular Prins reaction (*i.e.*, **4**→**5**), and the formation of the heterocyclic F-ring (*i.e.*, **5**→**6**). Although ring closure of the G-ring did not occur under the conditions used, brief exposure of **6** with the acidic resin Amberlyst-15^®^ produced perovskone in 90% yield.

We recently reported simpler conditions whereby quinone (*S*)-**2** reacted with triene **3** to directly provide (+)-perovskone (**1**) in which five bonds, four rings, and six chiral were created in a single operation [42,43]. Please note the mild conditions employed in this one-pot transformation: 0 °C for ninety minutes followed by warming at 50 °C for seven h.

**Scheme 3:**
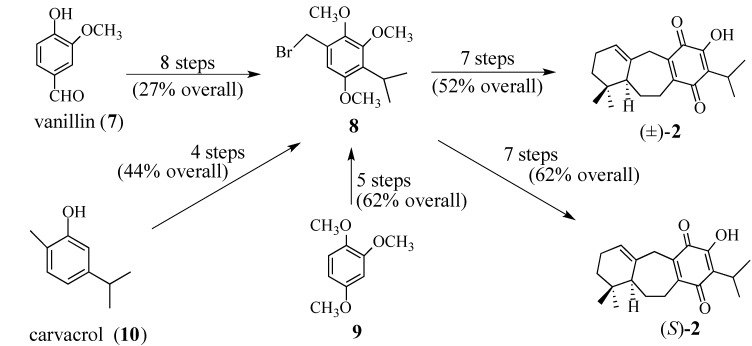
The preparation of quinone (±)-**2** and (*S*)-**2**.

In our initial synthesis of **1** vanillin (**7**) was converted in eight steps to bromide **8** in 27% overall yield [38]. The subsequent conversion of **8** to quinone (±)-**2** required seven additional steps and occurred in 52% overall yield (Scheme 3). The use of 1,2,4-trimethoxybenzene (**9**) [44,45] or carvacrol (**10**) as the starting material [46] to prepare bromide **8** required fewer steps and gave better overall yield, without the isolation or purification of any intermediates. Since the C-5 chiral center in quinone **2** controls the stereochemistry for each new chiral center produced in the tandem polycyclizations leading to (+)-perovskone, the preparation of quinone **2** in optically active form was essential to prepare (+)-perovskone. Quinone (*S*)-**2** was prepared from bromide **8** in seven steps and 62% overall yield [45]. 

## 2. Results and Discussion

“Many syntheses only appear as terse communications in journals. Very rarely do chemists discuss the blind alleys and dead ends that were encountered in a synthesis. This is unfortunate for the student who wants to learn about synthesis. I think many students have the mistaken impression that organic chemists conceive a brilliant ‘paper’ synthesis in 1 h and hand it over to their graduate students who see it through to completion without any problems or difficulties. However, in almost every synthesis there are problems to be overcome and obstacles to be surmounted.”

[47]

This review summarizes our exploration of the Diels-Alder reactions of 12-hydroxy-9(10→20)-5aH-abeo-abieta-1(10),8(9),12(13)-triene-11,14-dione, which will henceforth be referred to as simply quinone **2** or quinone (*S*)-**2**, for the synthesis of perovskone and related natural products.

### 2.1. The Perovskone Diels-Alder Reaction

Quinones are excellent dienophiles for Diels-Alder reactions [48,49]. Nevertheless, the Diels-Alder reaction between quinone (*S*)-**2** and any diene presents four fundamental questions that must be answered:

- Will the cycloaddition be facially selective?
- Will the diene component be stable under the Diels-Alder reaction conditions employed?
- Will the Diels-Alder adduct be stable under the Diels-Alder reaction conditions used?
- Will the cycloaddition be regiospecific?

The facial selectivity of the Diels-Alder reaction of quinone **2** is addressed in Section 2.1.1. The three remaining questions are discussed in Section 2.1.2.

#### 2.1.1. Facial Selectivity of Quinone **2**

**Figure 1:**
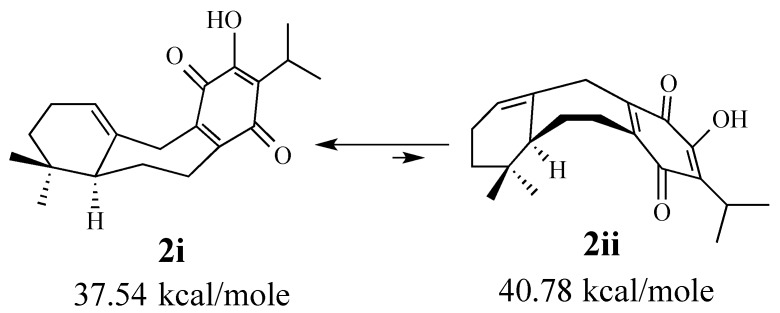
MM3 calculations of two conformations of quinone **2**.

MM3 calculations [50] indicated that the chair cycloheptene conformer **2i** is 3.4 kcal/mole lower in steric energy than the boat conformation **2ii** (Figure 1). The magnitude of this energy difference suggests that the equilibrium between these conformers would strongly favor the cup-shaped conformation. The Diels-Alder reaction featured in our synthesis of (±)-perovskone gave only the adduct derived from addition to the α-face of (±)-**2**, which established the correct relative configurations at C-8, C-9, and C-24 (cf. adduct **4**). 

#### 2.1.2. Other Dienes Studied

**Scheme 4:**
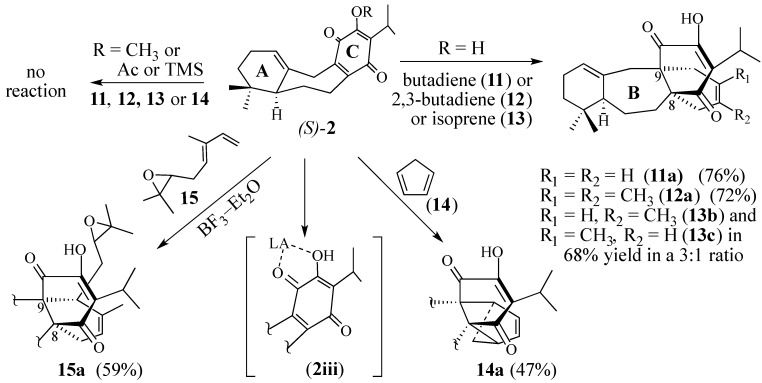
Diels-Alder reactions of (*S*)-**2**with five simple dienes.

The Diels-Alder reactions of functionalized quinones with functionalized dienes under thermal conditions generally fail [51]. Surprisingly, quinone **2** undergoes Diels-Alder reaction slowly at room temperature with reactive dienes such as 1,3-butadiene (**11**), 2,3-dimethylbuta-1,3-diene (**12**), isoprene (**13**) and cyclopentadiene (**14**), but reacts more rapidly when heated (Scheme 4). Common techniques used to overcome a sluggish Diels-Alder reaction are to carry out the cycloaddition either in the presence of a Lewis acid catalyst [52] or under high pressure [53,54,55], or a combination of both conditions [56]. Quinone **2** reacts cleanly with epoxide **15**, the result of treating mycrene with *m*-CPBA, over a ten-hour period at 25 °C to produce adduct **15a**, despite the use of BF_3_–Et_2_O, a much stronger Lewis acid than Eu(fod)_3_. This result gave us confidence that the Diels-Alder reaction with **3** would be regiospecific. Surprisingly, changing the C-12 hydroxyl group to either a methyl ether, an acetate, or a silyl ether caused the protected dienophiles to resist Diels-Alder reaction with reactive dienes **11**–**14** even in the presence of Lewis acid catalysts. 

**Scheme 5:**
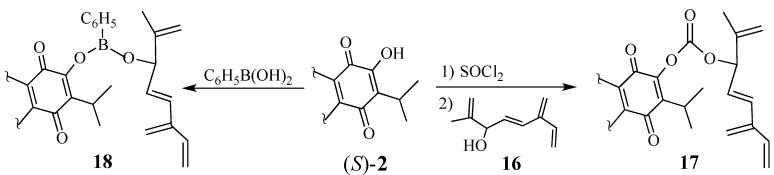
Intramolecular Diels-Alder reactions to make the triterpene skeleton.

The tethering of the dienophile to the diene moiety often facilitates Diels-Alder reactions and ensures the stereochemical outcome of the cycloaddition [56,57,58,59]. However, in our hands, linking quinone (*S*)-**2** with allylic alcohol **16**, either as a carbonate (cf. **17**) or as a phenylboronic acid ester (cf. **18**) [60], failed to achieve the desired [4+2]-cycloaddition either thermally or with Lewis acid activation (Scheme 5) [61].

Several important observations were made while studying the Diels-Alder reaction of dienes **11**–**15**. First, most Diels-Alder reactions required at least 10 mol % of the Lewis acid relative to the quinone. We believe that complexation between the Lewis acid and the α-hydroxyl group and the C-11 carbonyl group of **2** (cf. chelate **2iii**, Scheme 4) is necessary for the Diels-Alder reaction to occur. This conjecture is supported by the work of Trost and co-workers [62] and Seebach and co-workers [63] who have proposed similar chelates in their Lewis acid-catalyzed Diels-Alder reactions of α-hydroxy- and α-methoxyquinones. Moreover, the effects of Lewis acids on Diels-Alder reactions of quinones, as well as the Diels-Alder reactions of quinones in water have been theoretically studied and support complexation activation [64,65,66]. In general, the Diels-Alder reactions were sluggish when carried out at 0 °C or at room temperature. Bidentate Lewis acids which can complex with both the dienophile and the diene moiety worked best. In theory, since the C-12 hydroxyl group of quinone **2** is a vinylogous carboxylic acid, it might catalyze the Diels-Alder reaction. However, simply heating **2** and **3** together at 200 °C gave only a trace amount of the [4+2]-adduct. 

#### 2.1.3. Modifications of the Dienophile

**Scheme 6:**
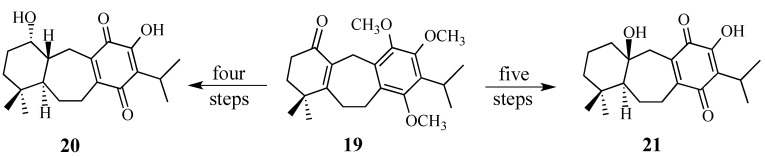
Modest modifications to quinone **2**.

Two modified dienophiles were investigated. Although alcohols **20** and **21** were readily available from enone **19**, a precursor to quinone **2**, these dienophiles were not stable to heating or to the presence of Lewis acids and therefore gave extremely low yields of the desired Diels-Alder adducts (Scheme 6). 

### 2.2. Studies Directed Toward the Synthesis of Salvadione-B and Salvadiol

**Scheme 7:**
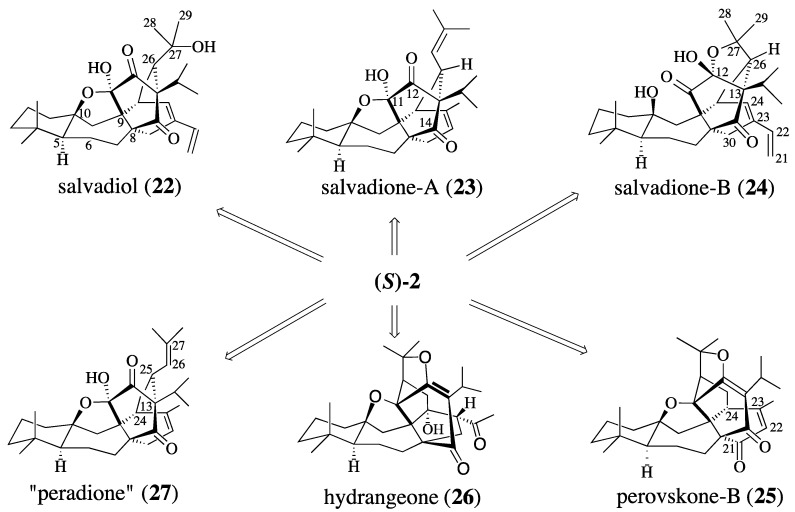
Six tritepenes.

Numerous anti-tumor and other bioactive constituents have been isolated from the various species of plants in the family Lamiaceae [67]. The genus *Salvia*, which comprises more than 800 species, is the largest genus of the Lamiaceae family. *Salvia bucharica*, popularly known in Pakistan as “susaudah,” is found throughout all of Central Asia [68,69,70] and is used for treating liver disorders and for its cooling effects. The isolation of salvadiol (**22**) [71], salvadione-A (**23**) [72] and salvadione-B (**24**) [72] from susaudah was reported in 1999 (Scheme 7) [73,74]. In 2011, (+)-perovskone-B (**25**) [75,76] and (+)-hydrangenone (**26**) [77] were isolated from *Salvia*. Scrutiny of salvadiol (**22**) and salvadione-B (**23**) reveals that these compounds are isomeric, differing only at the chirality of C-26, and whether C-11 or C-12 is a hemi-ketal. Since salvadiol, salvadione-A, and salvadione-B possess many of the salient features of perovskone [78], we were confident that they could be synthesized from quinone (*S*)-**2** via biomimetic cascade reactions. A selective allylic oxidation of the d-ring of perovskone would produce perovskone B, whereas hydrangenone (**26**) may be derived from **1** by cleaving the D-ring, followed by an aldol condensation. Finally, we believe that the published structure of peradione, elucidated using only NMR experiments [79], is incorrect. We intend to resolve this ambiguity through the synthesis of our proposed structure for “peradione” (cf. **27**). Nevertheless, quinone (*S*)-**2** is the key intermediate for the synthesis of salvadiol, salvadione-A, salvadione B, as well as other members of this family of triterpenes such as perovskone-B and hydrangenone. This potential of quinone(*S*)-**2** to serve as a common precursor for several complex natural products has inspired others to also prepare (±)-**2** via alternative strategies [80].

#### 2.2.1. Epoxide-Based Synthesis of Salvadione-B (**24**) and Salvadiol (**22**)

**Scheme 8:**
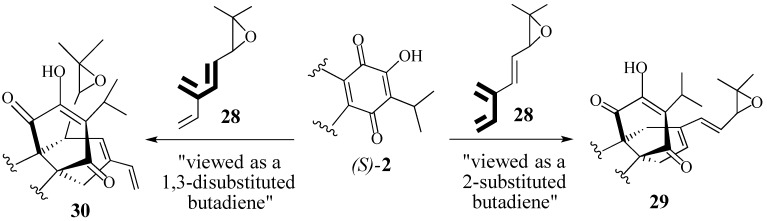
Triene **28** has regioselecitivity issues.

The Diels-Alder reaction of (*S*)-**2** with epoxy triene **28** is the key step in our routes to synthesize both *Salvia* metabolites (Scheme 8). 

Prior work in our labs has established that cycloadditions to quinone (*S*)-**2** occur only from the α-face and are regiospecific. However, the use of conjugated triene **28** as the diene component raises an interesting, albeit serious, question about the regioselectivity of the Diels-Alder reaction: Will triene **28** react as a 2-substituted butadiene to give adduct **29** or will it react as a 1,3-substituted butadiene? Further analysis reveals that both dienes moieties can adopt an *s*-cisoid conformation; steric interactions neither rule out, nor favor, either possibility. Based on electronic effects, the more substituted butadiene is assumed to be more reactive which would give adduct **30**. However, an epoxide is an electron-withdrawing substitute which might have a negative effect on the reactivity of the 1,3-disubstituted butadiene. These considerations make the regioselectivity of this Diels-Alder reaction difficult to predict and can only be answered experimentally. 

**Scheme 9:**
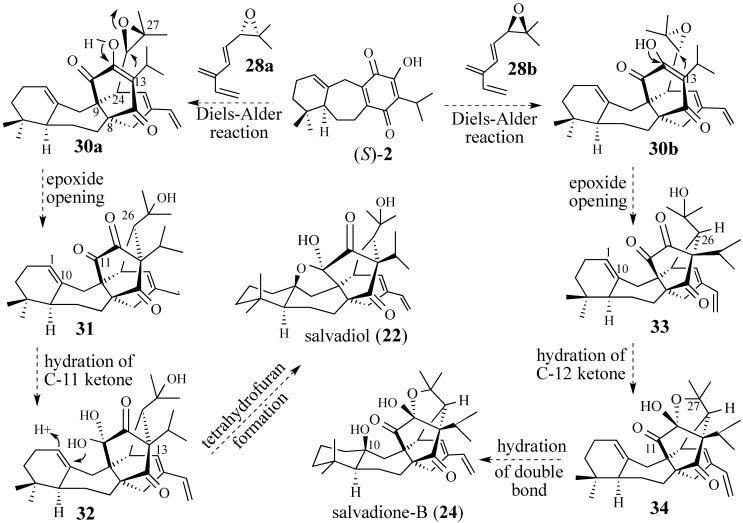
Epoxide-based retrosynthetic analysis for salvadione-B (**24**) and salvadiol (**22**).

After the Diels-Alder reaction (cf. **30a**/**30b**), three additional transformations were envisioned for the synthesis of **22** and **23** (Scheme 9). Note that epoxy triene **28a** culminates in a synthesis of salvadiol (**22**), whereas its enantiomer **28b** permits a short synthesis of salvadione-B (**24**). For convenience sake we will first focus on our proposed synthesis of salvadiol.

Epoxides are effective alkylating agents for 1,3-diketones [81]. The second step envisioned in our route to salvadiol is the intramolecular opening of the C-26, C-27 epoxide by the latent 1,3-dione moiety present in the Diels-Alder adduct (cf. **30a** → **31**). Since epoxides open in an *anti* fashion, the formation of the C-13, C-26 carbon–carbon bond sets the requisite stereochemistry at C-13 and at C-26. The intramolecular nature of this reaction dictates that epoxide opening can only occur at the less substituted C-26 position to form a six-membered ring. Hydration of the C-11 carbonyl of **32**, followed by the addition of one of the hydoxyl groups of hydrate **32** to the C-1, C-10 trisubstituted double bond would complete a synthesis of salvadiol (**22**). Our proposed synthesis of salvadione-B (**24**) parallels the strategy anticipated to synthesize salvadiol, except that epoxide **28b**, the enantiomer of **28a**, will be used in the Diels-Alder cycloaddition to produce adduct **30b**. As before, intramolecular epoxide opening by the 1,3-dione moiety in **30b** leads to the formation of tertiary alcohol **33** and fixes the stereochemistry at C-13 and C-26. Scrutiny of models of **33** reveals that the tertiary alcohol at C-27 is very close to the C-12 carbonyl, thereby permitting the formation of cyclic hemiacetal **34**. Further treatment of **34** with aqueous acid should hydrate the C-1, C-10 double bond to afford salvadione-B (**24**). In intermediate **34** the C-11 carbonyl and the hydroxyl group at C-10 are in the same plane, which precludes an additional tetrahydrofuran ring from forming.

Our cascade-based synthesis of perovskone suggests that the Diels-Alder reaction, the opening of the epoxide moiety by the latent 1,3-dione and the requisite carbonyl, and double bond hydrations may be achieved under Lewis acid-catalyzed conditions in a one-pot operation. If so, the reaction of racemic triene **28** with quinone (*S*)-**2** under such optimized conditions would directly produce salvadione-B and salvadiol. While this cascade-based transformation is a worthy goal, we decided to first prepare these complex triterpenes in a step-wise fashion before advancing a cascade-based strategy. Nevertheless, both strategies require the preparation of epoxy triene (±)-**28** and/or optically active epoxides** 28a** and** 28b** [82,83].

**Scheme 10:**
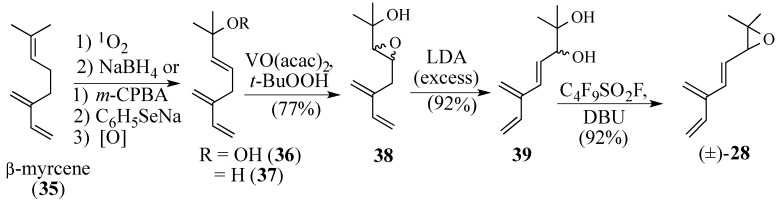
Preparation of epoxytriene **28**.

An attractive starting material for synthesizing epoxy triene **28** is β-myrcene (**35**) which has the complete carbon skeleton of **28** and one of the conjugated diene units (Scheme 10). The reaction of β-myrcene with singlet oxygen, followed by* in situ* reduction of the hydroperoxide **36** with sodium borohydride, provided allylic alcohol **37** in 50% yield [84,85]. We preferred to prepare allylic alcohol **37** on a 5-gram scale by selectively epoxidizing myrcene with *m*-CPBA, followed by opening of the trisubstituted epoxide with sodium phenylselenide and then eliminating the selenoxide intermediate (56% over three steps) [86]. Sharpless has shown that vanadium catalysts can selectively oxidize the double bond of an allylic alcohol, even in the presence of other double bonds [87]. Thus, epoxidation of **37** using VO(acac)_2_ produced epoxide **38**, which upon treatment with LDA at 0 °C, gave diol **39** in 70% yield over two steps. The stereochemistry of the C-3 secondary alcohol can be introduced via an enantioselective Sharpless epoxidation of **37** [88,89]. Although traditional means of converting the diol **39** into epoxy triene **28** failed [*i.e.*, MsO-, TsO-, or Mitsunobu reactions], perfluorobutanesulfonyl fluoride in the presence of DBU [90] gave epoxy triene **28** in 92% yield. Epoxide **28** was stable in THF, toluene and DCM at temperatures below 50 °C, but polymerized when heated above 50 °C. While **28** is stable to mild europium Lewis acids for more than two days at 25 °C, these conditions did not promote a Diels-Alder reaction with quinone **2**. We were disappointed to learn that regardless of the Lewis acid used, or the conditions employed, the hoped for Diels-Alder reaction was not observed. 

**Scheme 11:**
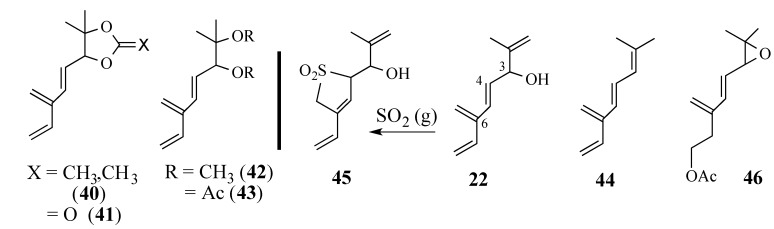
Derivatives of triene diol **39**.

Derivatives **40**–**43** were ready prepared from diol **39** but none of these trienes reacted with quinone **2** (Scheme 11). Tetraenes **22** and **44** were prepared and treated with quinone **2** without success. Interestingly, treatment of **22** with SO_2_ gave adduct **45** corresponding to the Diels-Alder addition to only the 4,6-diene moiety. Epoxy diene **46** was prepared in which the terminal double bond was masked as an acetate, which could be eliminated later in the synthesis after the Diels-Alder reaction had occurred. Inexplicably, diene **46** failed to undergo either thermal or Lewis acid-catalyzed Diels-Alder reaction with quinone **2**.

**Scheme 12:**
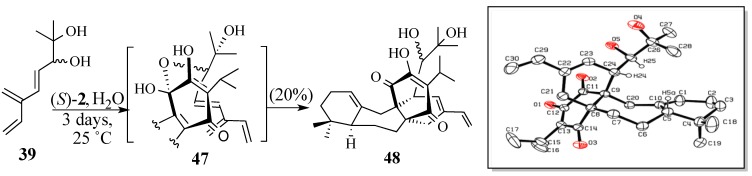
The aqueous Diels-Alder reaction of triene diol **39**.

In contrast, triene diol **39** underwent Diels-Alder reaction with quinone **2** using water as the reaction medium [91,92] in a 20% yield of the Diels-Alder adduct **48** (Scheme 12); an X-ray analysis of adduct **48** confirmed the predicted facial and regiospecificity of this cycloaddition [93]. We speculated that the* in situ* formation of hemi-ketal intermediate **47** causes an intermolecular cycloaddition to become an intramolecular one which controls the regiospecificity of the Diels-Alder reaction. Unfortunately, this aqueous cycloaddition could not be optimized because of the poor solubility of quinone **2** in water. The use of water-soluble co-solvents, such as THF or dioxane, did not improve the reaction yield; nor did adding weak bases, such as sodium bicarbonate or sodium hydroxide, to improve the solubility of the quinone [94].

**Scheme 13:**
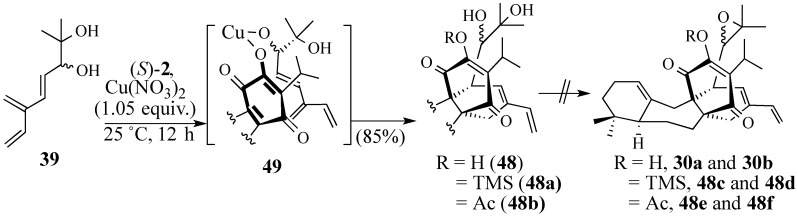
Copper nitrate-catalyzed Diels-Alder reaction of triene diol **39**.

Tethering quinone **2** and triene diol **39** together using a bidentate Lewis acid (cf. **49**, Scheme 13) in trifluoroethanol [95] produced tetracycle **48** in 85% yield. Unfortunately, all attempts to convert diol **48** into epoxides **30a**/**30b** failed, presumably because of the acidity of the C-12 hydroxyl group. The TMS ether **48a** and acetate **48b** were readily prepared from **48**. However, the diol moieties of **48a** or **48b** could not be converted into epoxides **48c/d** and **48e/f**, respectively. Thus, we concluded that a new strategy was needed to form the C-13, C-26 sigma bond.

#### 2.2.2. An Alternative Diels-Alder Strategy to Synthesize Salvadione-B (**24**)

**Scheme 14:**
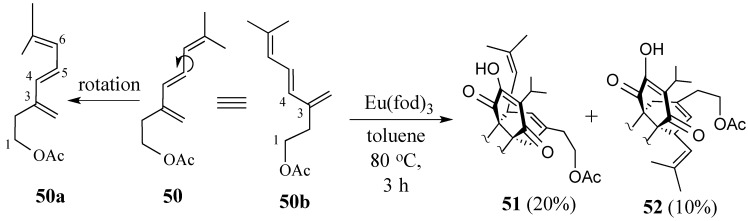
The Diels-Alder reaction of (*S*)-**2** and triene acetate **51**.

Our revised strategy to prepare salvadione-B used triene acetate **50** in which the electron-withdrawing epoxide was replaced by a double bond (Scheme 14) giving two set of dienes that could take part in the cycloaddition. The presence of a *Z-*methyl substituent as part of the 4,6-diene moiety would reduce its reactivity by hindering the likelihood of the *s-cis* conformer **50a**. In contrast, rotation about the C-5, C-6 sigma bond allows the 3,4-diene to easily adopt a *s-cis* conformation without severe steric interactions (cf. **50b**).

When the reaction between triene acetate **50** and quinone (*S*)-**2** was carried out in toluene at 80 °C for 3 h with a catalytic amount of Eu(fod)_3_, two major products were produced along with mostly unreacted quinone and triene. The less polar product was identified as the desired Diels-Alder adduct **51** (20%) while the more polar product, which was found to have similar spectra and same molecular weight to the desired adduct, was identified as the regioisomer **52** (10%). Conditions have been optimized so that only adduct **51** is produced in 70% yield. In 2009, cycloaddition adduct **51** was converted to salvadione-B (**24**) both in a stepwise fashion and via two consecutive one-pot operations [73,74].

### 2.3. Synthesis of (+)-Salvadione-A (**23**)

(+)-Salvadione-A (**23**), which has six rings and eight chiral centers, was synthesized from quinone (*S*)-**2** in four steps featuring a facial and regiospecific Diels-Alder reaction.

#### 2.3.1. Diels-Alder reaction of triene ether **53** and quinone (*S*)-2

**Scheme 15:**
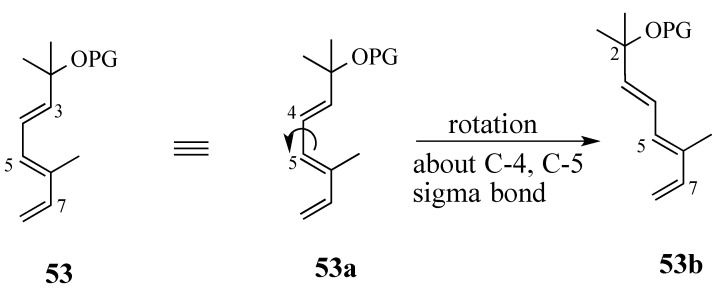
Comformational analysis of triene acetate **54.**

The regiochemical outcome of the Diels-Alder reaction of triene **53** was easy to predict (Scheme 15). Scrutiny of triene **53** indicates that the presence of a *Z*-methyl substitutent as part of the 3,5-butadiene moiety hampers the formation of *s*-*cis* form **53a** whereas the 5,7-diene moiety does not suffer such a steric effect (cf. **53b**). Hence, the cycloaddition of quinone **2** with triene **53** was expected to involve only the 5,7-diene unit.

**Scheme 16:**
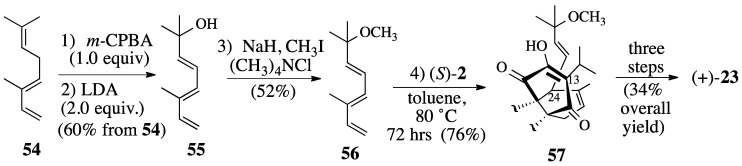
The Diels-Alder reaction between quinone (*S*)-**2** and triene ether **56**.

Epoxidation of trans-β-ocimene (**54**) with *m*-CPBA, followed by treating the intermediate epoxide with excess LDA, gave (3*E*,5*E*)-octa-3,5,7-trien-2-ol (**55**) in 60% overall yield (Scheme 16). Although tertiary alcohol **55** could be protected as an acetate, this acetate undergoes rapid decomposition at ambient temperature or upon attempted chromatography on silica gel. Methyl ether **56** was prepared in the hope that the methoxy group would be less prone to elimination. 

Indeed, triene ether **56** was thermally stable and reacted with quinone (*S*)-**2** at 80 °C over a 72-hour period to afford Diels-Alder adduct **57** in 76% yield. A discussion of the conversion of Diels-Alder adduct **57** to salvadione-A (**23**) can be found elsewhere [43,45].

#### 2.3.2. The Microwave-Promoted Diels-Alder Reaction of triene ether **56** and (*S*)-2

In 1986, Gedye and co-workers reported that hydrolysis reactions and some oxidations benefitted from microwave irradiation [96,97]. Soon afterwards, Giguere and Majetich reported their independent observations that Diels-Alder, Claisen, and ene reactions all demonstrated significant rate enhancements when compared to traditional heating methods [98,99,100,101]. A search of the terms “microwaves” and “synthesis” using *SciFinder Scholar* on May 23, 2013 produced a list of 64,739 references that have been published in the past twenty-seven years. It is now commonplace to find microwave systems in academic, industrial, and hospitals settings for the rapid synthesis of radioisotope-labeled drugs. Thus, it is not surprising that we would investigate the use of microwave irradiation to promote the Diels-Alder reactions of quinone **2**. 

**Scheme 17:**
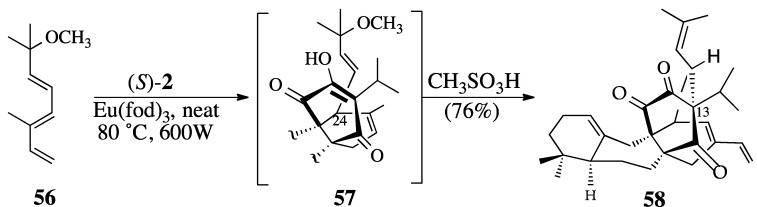
Tandem microwave-promoted Diels-Alder of **56** and S_N_2' displacement.

Initially, we used water as the reaction medium or as a co-solvent because it couples very well with microwave irradiation. For example, the Diels-Alder reaction of triene acetate **50** to form cycloaddition adduct **51** was achieved using microwave heating (cf. Scheme 14). However, further study showed that this Diels-Alder reaction worked best under microwave heating without any solvent. The microwave-promoted Diels-Alder reaction of triene **56** and quinone (*S*)-**2** neat produced Diels-Alder adduct **57** in good yield (Scheme 17). Addition of a catalytic amount of methanesulfonic acid dissolved in THF to the crude Diels-Alder adduct **57** gave a 76% yield of **58** the Diels-Alder/displacement product. Since Teflon reactions vessels were used [a *Milestone Inc.*, Ethos One system], these observations suggest that one of the Diels-Alder components may be absorbing the microwave irradiation and thus thermally promoting the cycloaddition. If true, this may be an example of the long sought for “magical microwave effect.” However, we are skeptical of this conclusion and intend to re-investigate this transformation.

### 2.4. Studies Directed toward a Synthesis of “peradione” (**27**)

**Scheme 18:**
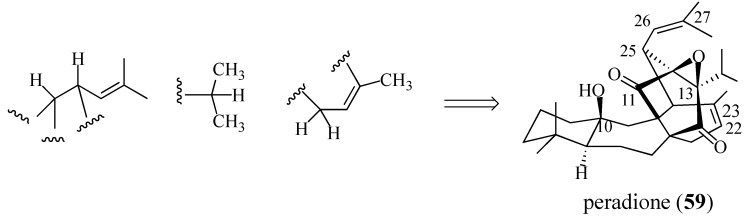
Key subunits of peradione based on NMR analysis.

In 1993, Ahmad and co-workers isolated the triterpene peradione (**59**) from *Perovskia abrotanoides*, another widely-used Pakistani medicinal plant (Scheme 18) [79]. The structure of this compound was elucidated by extensive spectroscopic studies and the following conclusions were made: (1) the molecular formula of peradione is C_30_H_42_O_4_; (2) ^13^C-NMR analysis indicated the presence of seven methyl groups, seven methylene units, six methines, and ten quaternary carbon atoms; (3) two ketones are present; (4) two double bonds are present, (5) five tertiary methyl groups are present; (6) two secondary methyl groups are present; and (7) three oxygen-bonded quaternary carbons (δ 70.1, 90.5, and 100.4) are present. In addition, ^13^C decoupled HMQC [Heteronuclear Multiple Quantum Coherence], 2D-COSY [Correlation Spectroscopy], HMBC [Heteronuclear Multiple Bond Coherence] and HOHAHA [Homonuclear Hartmann-Hahn] experiments indicated the presence of three structural subunits (shown below). Based on this information, peradione was assigned structure **59**.

#### 2.4.1. A Wrong Structural Assignment

**Scheme 19:**
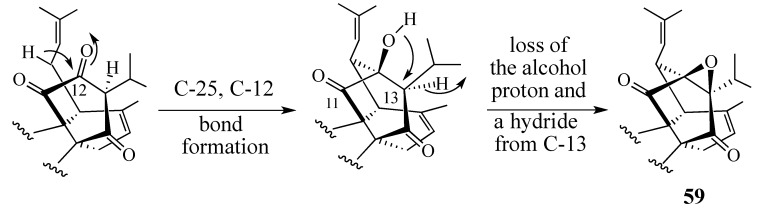
The proposed biogenetic synthesis of peradione.

While modern spectroscopic techniques have greatly facilitated structural determination, errors do occur. For example, it is very difficult to make a Dreiding molecular model of the structure corresponding to compound **59**, and its proposed biogenetic pathway requires a highly unlikely epoxidation sequence (Scheme 19). The researchers who assigned peradione this structure and suggested the biogenetic pathway were informed of our misgivings. To date, despite repeated requests, we have been denied copies of their NMR data or an authentic sample of peradione (**59**) to carry out our own structure determination.

Our proposed structure for “peradione” (*i.e.*, **27**, Scheme 7) benefits from the isolation of salvadione A in 1999 and its characterization by single-crystal X-ray diffraction analysis [33]. Comparison of the ^13^C-NMR data of peradione with that of perovskone (**1**), salvadione-A (**23**), savadione-B (**24**), and salvadiol (**22**) reveals many similiaries. Most of the differences in these compounds lie in the connectivities of C-25 with either C-12 or C-13, and the oxidation state of C-11 or C-12. The published structure for peradione (cf. **59**) has a C-11 carbonyl, a C-12, C-13-epoxide, and a tertiary alcohol at C-10. This structure is based on the interpretation that the δ 70.1, 90.5, and 100.4 quaternary carbons atoms are bonded to oxygen. We believe that these signals better correspond to a hemiacetal linking C-10 and C-11 (δ 90.5 and 100.4, respectively) and that the δ 70.1 signal corresponds to a quaternary carbon bearing the isopropyl group and positioned between the two ketones. We therefore believe that peradione is actually the C-25 epimer of salvadione-A (**23**). This belief allows us to suggest an expedient route to synthesize salvadione-A and validate our proposed structure for “peradione”.

#### 2.4.2. A Displacement-Based Strategy to Prepare Salvadione-A (**23**) and Our Proposed Structure for Peradione (**27**)

**Scheme 20:**
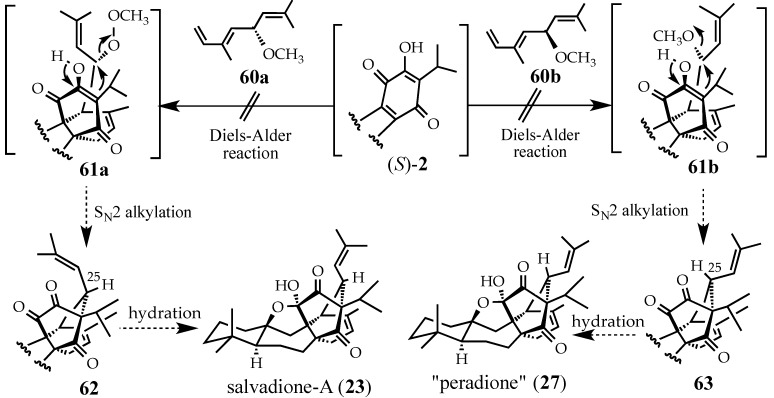
Proposed syntheses of “peradione” (**27**) and salvadione-A (**23**).

We believed that the Diels-Alder reaction of quinone (*S*)-**2** with methyl ether **60a** would occur at temperatures comparable to those used in our stepwise salvadione-A (**23**) synthesis (Scheme 16). The addition of Lewis acid to crude Diels-Alder adduct **61a** would also promote the subsequent intramolecular S_N_2-alkylation (cf. **62**, Scheme 20), and may aid in the sequential hydrations of the C-11 carbonyl and tetrahydrofuran formation. Ideally, the use of methyl ether **60b** will produce cycloaddition adduct **61b**, followed by the S_N_2-alkylation to produce trione **63** which should culminate in a synthesis of “peradione” (**27**).

(±)-Ether **60** was prepared via a Williamson ether synthesis from 2,6-dimethyl-2,5,7-octatrien-4-ol, a constituent of two narcissus varieties [102]. Unfortunately, in our hands, ether **60** was extremely sensitive and decomposed faster than it reacted in Diels-Alder fashion. Although conceptually attractive, this strategy to prepare salvadione-A (**23**) and “peradione” (**27**) was abandoned.

## 3. Conclusions

We have found that quinone (*S*)-**2** undergoes Diels-Alder reactions which has facilitated the efficient syntheses of perovskone (**1**), salvadione-A (**23**) and salvadione-B (**24**). Unfortunately, the Diels-Alder reactions studied to prepare salvadiol (**22**) and “peradione” (**27**) failed because the diene component rapidly decomposed under the experimental conditions investigated. Nevertheless, we are confident these natural products will one day be synthesized using quinone (*S*)-**2** and feature other Diels-Alder reactions of more stable dienes. We also believe that the genus *Salvia* will continue to yield new triterpenes structurally related to the perovskones and/or the salvadiones; thereby increasing the likelihood that quinone (*S*)-**2** may be featured in future synthetic work.
